# Ovarian stimulation toward oocyte cryopreservation for fertility preservation in a patient with Hirata syndrome: a clinical challenge in assisted reproduction

**DOI:** 10.1007/s00404-025-08245-7

**Published:** 2026-02-06

**Authors:** Konstantinos Karkalemis, Nektaria Papadopoulou-Marketou, Emmanouil Kalampokas, Maria Simopoulou, Theodoros Kalampokas

**Affiliations:** 1 Second Department of Obstetrics and Gynaecology, “Aretaieio” University Hospital, Medical School, National and Kapodistrian University of Athens, Athens, Greece; 2 First Department of Propaedeutic and Internal Medicine, Medical School, National and Kapodistrian University of Athens, Athens, Greece; 3 Department of Physiology, Medical School, National and Kapodistrian University of Athens, Athens, Greece

**Keywords:** Insulin autoimmune syndrome (IAS), Hirata disease, Assisted reproductive technology (ART), Autoimmune hypoglycemia, Oocyte cryopreservation

## Abstract

**Background:**

Insulin autoimmune syndrome (IAS), or Hirata disease, is a rare autoimmune disorder characterized by the presence of autoantibodies targeting insulin, leading to episodes of postprandial hypoglycemia. First identified in Japan, the condition was historically seen primarily in the Asian population, but with global recognition and improved diagnostic tools, its prevalence has expanded. While IAS is often self-limiting and resolves with dietary modifications and discontinuation of triggering medications, its management in the context of assisted reproductive technology (ART) remains understudied.

**Case Presentation:**

This case report discusses a 28-year-old female diagnosed with IAS who underwent oocyte cryopreservation following a fertility assessment revealing low serum AMH levels. Despite a history of severe hypoglycemia, which was managed with rituximab and resolved within a month, and the presence of elevated insulin and insulin autoantibodies, she successfully completed ovarian stimulation without experiencing hypoglycemic episodes.

**Discussion:**

Close monitoring of glucose levels and insulin autoantibody concentrations was essential for successful oocyte retrieval. This case underscores the importance of careful monitoring and individualized care for patients with IAS undergoing ART as autoimmune flare-ups and hypoglycemia can still occur even when the disease is in remission.

**Conclusion:**

A multidisciplinary approach involving reproductive endocrinologists and fertility specialists is critical for safe management of such patients.

## What does this study add to the clinical work?

**Table:** 

To our knowledge, this is the first reported case of ovarian stimulation in a patient with IAS. This article further supports that both the diagnosis of IAS but mostly the appropriate endocrinological management can ensure a safe and successful fertility preservation process without hypoglycemic events, while research continues to clarify the pathophysiology of the disease.	

## Introduction

Insulin autoimmune syndrome, also known as Hirata disease, is a rare autoimmune disorder which exhibits episodes of spontaneous postprandial hypoglycemia and is characterized by the presence of autoantibodies targeting insulin [1]. It was first described in Japan in 1970 by Hirata et al. and for a lot of years was considered a syndrome mainly affecting the Asian population [2]. However, worldwide use of triggering medications as well as better diagnostic tools has led to reports of the disease with broader prevalence worldwide [3].

Insulin autoimmune syndrome has been classified as idiopathic or drug induced according to the presence or absence of a triggering factor in the form of medication or viral infection [4, 5]. Most common medications associated with insulin autoimmune syndrome are antithyroid drugs, especially methimazole, often used for the treatment of Graves’ disease, as well as a list of other common drugs including antihypertensives, protein pump inhibitors, antibiotics and steroids [4]. In addition, drugs containing sulfhydryl groups, like alpha-lipoid acid supplements have been associated with the production of insulin autoantibodies [4, 6].

Insulin autoimmune syndrome is further classified as classic, when the production of autoantibodies isn’t linked to exogenous insulin administration and to non-classical when the clinical manifestation of IAS happens following use of insulin analogs [5].

The clinical manifestations of IAS vary from mild symptoms of hypoglycemia to seizures and coma [7]. The disease usually follows a biphasic pattern of early postprandial hyperglycemia which progresses to postprandial hypoglycemia [6]. As described by Capellani et al. [8], post-meal hyperglycemia causes the normal pancreatic stimulation for the secretion of insulin. However, the circulating insulin is immediately bound to the circulating autoantibodies blocking its ability to counter hyperglycemia. Pancreatic cells continue to further secrete insulin as hyperglycemia persists. The insulin autoantibody complexes later dissolve spontaneously, even at times when glucose concentrations have returned to normal or are even low, leading to a sudden excess of circulating insulin which eventually causes hypoglycemia.

After excluding exogenous insulin analogs or other hypoglycemic drugs, the diagnostic work-up then requires distinguishing IAS from two other conditions that present with hypoglycemia, the insulinoma and the type B insulin resistance [4, 6]. Insulin levels are calculated, which are generally very high in IAS, often more than 1000 μ/mL while in insulinoma, insulin levels don’t rise to that extent. C-peptide levels are also measured for the exclusion of exogenous insulin administration. The ratio of insulin/C-peptide > 1in IAS due to the excessive insulin production and despite the higher, in comparison, half-life of C-peptide [9]. Insulinoma, if not suspected by CT of the pancreas, usually doesn’t reverse the normal ratio of insulin/C-peptide of < 1 [3, 8]. Definite diagnosis of IAS requires the detection of insulin autoantibodies. Table 1 provides an overview of distinguishing laboratory and imaging featured that are commonly associated with each hypoglycemic condition.

**Table 1:** Differential diagnosis of hypoglycemia based on hormonal testing and pancreas CT imaging

	Insulin autoimmune syndrome (IAA)	Insulinoma	Type B insulin resistance	Exogenous insulin administration
Insulin	Very high (> 1000 μ/L)	High (but < 1000 μ/L)	High	High (C-peptide low)
Insulin/C-peptide ratio	> 1	< 1	< 1	> 1
Autoantibodies	IAA+	(−)	IAA (−) Anti-insulin receptor (+)	(−)
CT scan	Normal	Suspicious lesion in pancreas	Normal	Normal

Patients with insulin autoimmune syndrome may present undiagnosed often due to the mild symptoms and self-limiting nature of the disorder. It’s because of this that the disorder mostly remains under the treating physicians’ radar and the existing literature about the management of patients with IAS undergoing ART therapy is scarce.

Due to its mild symptoms and self-limiting nature, insulin autoimmune syndrome (IAS) may go undiagnosed in patients presented to infertility clinics for assisted reproductive techniques, often escaping the attention of treating physicians.

As a result, our knowledge regarding the management of patients of IAS undergoing ART is limited, with no prior case reported in the literature about how and whether the hormonal fluctuations during stimulation, and the excess of estrogens, could affect the glycemic control in these patients.

Here we present a rare case of a woman with diagnosed IAS undergoing ovarian stimulation for fertility preservation purposes. We decided on and suggest frequent monitoring of glucose and insulin autoantibody levels, in consultation with an endocrinologist for optimal results.

## Case presentation

A 28-year-old female Cypriot patient with IAS who was referred to our outpatient office for desired oocyte cryopreservation after the identification of low serum AMH levels (0.29 ng/mL) in a fertility work-up. The patient expressed a preference for delayed childbearing and opted for oocyte cryopreservation. She had no history of previous pregnancy (G0) or history of any surgery. Apart from a sedentary lifestyle and smoking, she didn’t have any other comorbidities. The age of menarche was 14 years old and she reported a regular menstrual cycle. There was no personal or family history of diabetes mellitus, other endocrinopathies or autoimmune disorders and she did not take any medication.

She was diagnosed with Hirata syndrome the previous year. In the onset of the disease, her symptoms were repeated episodes of severe hypoglycemia and she was hospitalized for work-up of the low blood sugar levels (often Glu levels were < 40 mg/dL several times daily). Along with severe hypoglycemia, abnormally high insulin levels and C-peptide (> 3000 mIU/L and > 7 ng/mL respectively) were observed, excluding exogenous administration. MRI of the abdomen as well as PET Gallium 68 demonstrated no insulinomas. Insulin autoantibodies test (IAA), which have a ligand unique for beta cells, was performed and a very high level was revealed (> 175 IU/mL). The patient was therefore diagnosed with Hirata syndrome and received dietary consultations. She received 1 dose of Rituximab and the episodes of hypoglycemia were resolved within a month. Since the time of diagnosis, the patient has been under continuous glucose monitoring (CGM) using the FreeStyle Libre system (Abbott) and received treatment with diazoxide at a dose of 25 mg daily. After 11 months of follow-up, diazoxide was discontinued as glycemic control was maintained without pharmacologic therapy. During the ART therapy work-up, on the transvaginal ultrasound, the antral follicle count (AFC) was assessed on day 5 of the menstrual cycle. The ultrasound revealed an AFC of 5 on the left ovary and AFC of 3 on the right ovary, indicating diminished ovarian reserve. FSH was 7.08 (reference range: 3.5–12.5 IU/L at follicular phase), LH was 8.36 mIU/L (reference range: 2.4–12.6 mIU/L at follicular phase), Estradiol (E2) was 14.3 pg/mL (reference range: 12.4–233.0 pg/mL at follicular phase), Prolactin (PRL) was 216 mIU/L (reference range: 102–496 mIU/L), and TSH was 2.44 mIU/L (reference range for < 65 years old: 0.270–4.2 mIU/L), all within normal range, suggesting a normal hormonal work-up. In addition, a low serum AMH 0.708 ng/mL (reference value 1.200–9.050 ng/mL for non-PCOS women under 29 years old) was identified. Further work-up included anti-ovarian IgG antibodies, which were negative and investigation for other autoimmune or endocrine disorders.

Although Hirata syndrome has been strongly associated with HLA-DR4 and specifically with the DRB1*0406 in the Asian population [8], HLA genetic testing was advised though the patient opted not to undergo further testing for financial reasons.

Meticulous glucose control was highly important during the whole process and insulin/IAA levels were assessed as summarized below in Table 2.

**Table 2:** Fasting insulin, C-peptide & IAA levels at diagnosis, before and 1 month after ovarian stimulation

	Fasting insulin	C-peptide	Insulin autoantibodies (IAA)	Glucose
SI units/reference range	2.6–24.9 mIU/L	0.8–4.4 ng/mL	< 20 IU/mL	mg/dL
At Hirata diagnosis	> 3000	7.12	> 175	< 35
Before ovarian stimulation	173	1.43	169	
1 month after ovarian stimulation	126	1.64	136	

The patient underwent two cycles of ovarian stimulation in June and July 2024 using the same protocol. The patient initially received 300 IU urofollitropin and r-hLH analog and on approximately the 10th day of stimulation, a GnRH antagonist was added, to prevent premature ovulation. Flexible antagonist protocol was used and the GnRH antagonist was initiated on the day 10, when the leading follicle reached 14 mm in diameter. Final oocyte maturation was achieved with 250 mcg of choriogonadotropin alpha when the appropriate follicle size criteria were met (follicle diameter of 18–20 mm), and oocyte retrieval was performed approximately 36 h later. A successful retrieval of 3 and 6 oocytes, respectively, was achieved. All 9 oocytes were mature (MII stage) and subsequently cryopreserved. At that time, the patient continued to exhibit elevated but gradually decreasing levels of fasting insulin (182.0 mIU/L; reference range: 2.6–24.9 mIU/L, ECLIA method) and insulin autoantibodies (IAA, 124 IU/mL). Despite these abnormalities, glycemic control remained adequate, and no hypoglycemic episodes were observed. During the subsequent six months, in addition to continuous glucose monitoring (CGM), the patient was regularly evaluated every three months for serum insulin, C-peptide, and insulin autoantibody (IAA) levels.

## Discussion

IAS is often a self-limiting disease that might remain undetected, and symptoms might dissolve without any intervention or medication. Usually, patients modify their dietary routine to avoid episodes of postprandial hypoglycemia by having frequent smaller meals and limiting monosaccharide intake. When diagnosed, IAS can be first approached by withdrawal of the triggering medication. Other therapies include glucocorticoids to counter antibody formation or other immunosuppressive agents like rituximab or azathioprine [5, 10, 11]. A-Glycosidase inhibitors, who prolong carbohydrate absorption in the GI tract and thus reduce early postprandial hyperglycemic episodes that stimulate the pancreas to produce excessive insulin. These measures often lead to full resolution within a few months, time required for the autoantibodies to disappear [5].

Ovarian stimulation and assisted reproductive technology (ART) can present unique challenges in patients with known autoimmune disorders. Specifically, in individuals with IAS undergoing ART, even those in clinical remission and not experiencing hypoglycemic episodes, hormonal therapy may represent a delicate balance. In women with autoimmune conditions such as systemic lupus erythematosus (SLE) or antiphospholipid syndrome (APS), ovarian stimulation has been associated with an increased risk of disease flare or increased disease activity [12]. Although the exact mechanisms are not fully understood, elevated estrogen levels during stimulation protocols are considered—particularly in genetically predisposed individuals—to potentially trigger autoimmune activity or flares [13]. However, due to the absence of direct evidence regarding how hormonal stimulation specifically impacts IAS, the response in these patients remains largely unpredictable. Further research is needed to better understand the safety and immunologic implications of ART in this patient population.

Reports of patients with IAS who successfully completed pregnancy have only emerged in the literature in very recent years, offering promising insights for the management of those undergoing ovarian stimulation—a population for whom data remain particularly scarce. Pregnancy by itself, has been proven to be a pathogenic factor of hypoglycemia in IAS, through a shift in cytokine balance favoring Th2 dominance that leads to insulin autoantibody production [14]. In one case, published by Viti et al., a 25-year-old pregnant woman was diagnosed with IAS at 24 weeks of gestation after experiencing hypoglycemic episode triggered by pregnancy supplementation that included α-lipoid acid. A combination of corticosteroids and discontinuation of the triggering supplement resulted in gradual diminishment of the hypoglycemic episodes and an uneventful delivery at 38 weeks [15]. In another case, IAS was diagnosed prior to the onset of the pregnancy, caused by medication for known Graves’ disease but resulted in an uneventful delivery with normal anti-insulin antibodies, blood glucose, and C-peptide levels throughout the pregnancy [16].

In our case, the patient, who was asymptomatic after treatment with rituximab one year earlier, successfully managed to complete the entire protocol of ovarian stimulation and oocyte retrieval with the purpose of oocyte cryopreservation without experiencing any episodes of hypoglycemia, thanks to close monitoring of glucose levels and the assessment of the patient's immune status throughout the process.

## Conclusion

Despite the self-limiting nature of the disease, patients with Hirata Syndrome undergoing ART therapy should be closed monitored during the whole process to avoid immune dysregulations that may result in hypoglycemic episodes. Given the complexity of the autoimmune disease, a multidisciplinary approach involving both reproductive endocrinologists and fertility specialists is crucial.

## Data Availability

No datasets were generated or analyzed during the current study.
